# Gafchromic XR‐QA2 film as a complementary dosimeter for hand‐monitoring in CTF‐guided biopsies

**DOI:** 10.1120/jacmp.v17i1.5725

**Published:** 2016-01-08

**Authors:** Sandra Sarmento, Joana Pereira, Maria José Sousa, Luís Cunha, Anabela G. Dias, Miguel F. Pereira, Augusto D. Oliveira, João V. Cardoso, Luís M. Santos, Margarida Gouvêa, Joana Lencart, João G. Alves, João A.M. Santos

**Affiliations:** ^1^ Centro de Investigação, Instituto Português de Oncologia do Porto Francisco Gentil (IPOPFG), E.P.E Porto Portugal; ^2^ Serviço de Física Médica, IPOPFG E.P.E. Porto Portugal; ^3^ Universidade de Lisboa (UL), Instituto Superior Técnico (IST), Laboratório de Protecção e Segurança Radiológica (LPSR) Bobadela LRS Portugal; ^4^ Serviço de Radiologia de Intervenção, IPOPFG E.P.E. Porto Portugal; ^5^ UL‐IST, Centro de Ciências e Tecnologias Nucleares (C2TN) Bobadela LRS Portugal; ^6^ Serviço de Radiologia, IPOPFG E.P.E. Porto Portugal; ^7^ Instituto de Ciências Biomédicas, Abel Salazar da Universidade do Porto Porto Portugal

**Keywords:** Gafchromic XR‐QA2 film, CTF‐guided biopsies, hand monitoring

## Abstract

Computed tomography fluoroscopy (CTF) is a useful imaging technique to guide biopsies, particularly lung biopsies, but it also has the potential for very high hand exposures, despite use of quick‐check method and needle holders whenever feasible. Therefore, reliable monitoring is crucial to ensure the safe use of CTF. This is a challenge, because ring dosimeters monitor exposure only at the base of one finger, while the fingertips may be exposed to the highly collimated CT beam. In this work we have explored the possibility of using Gafchromic XR‐QA2 self‐developing film as a complementary dosimeter to quantify hand exposure during CTF‐guided biopsies. A glove used in a previous study and designed to contain 11 TLDs was adapted to include Gafchromic strips 7 mm wide, covering the fingers. A total of 22 biopsies were successfully performed wearing this GafTLD glove under sterile gloves, and the IR reported no difficulty or reduction of dexterity while wearing it. Comparison of dose distributions obtained from digitization of the Gafchromic film strips and absolute $H_{p}$(0.07) readings from TLDs showed good agreement, despite some positional uncertainty due to relative movement. Per procedure, doses at the base of the ring finger can be as low as 3%–8% of hand dose maximum. Accumulated dose at the base of the ring finger was four times lower than the dose maximum.

PACS numbers: 07.57.Kp, 29.40.‐n, 85.25.Pb, 87.57.qp

## I. INTRODUCTION

CTF, also known as CT‐fluoroscopy or CT‐fluoro, is essentially computed tomography (CT) with the possibility of in‐room, real‐time imaging. Both CT and CTF can be used to guide interventional radiology procedures, allowing good visualization of small lesions and neighboring critical structures, as well as the projected needle pass. The real‐time imaging of CTF is particularly useful in lung biopsies, where respiratory motion often causes lesion displacement.^(1)^

Typical dose rates in CTF are much higher than in conventional fluoroscopy,^(2)^ and the biopsy needle is usually in the imaged plane. To reduce hand exposure to acceptable levels, two solutions have been proposed: the use of needle holders during continuous viewing,^1^, ^3^, ^4^, ^5^ and the quick‐check method of Silverman et al.^(6)^ whereby intermittent imaging is used to check needle position, but needle advancement occurs only during beam‐off.

Both solutions have some disadvantages. With the quick‐check method, the Interventional Radiologist (IR) cannot take full advantage of real‐time viewing, while the use of needle holders can result in loss of grip and tactile feedback.^6^, ^7^ During difficult procedures, IRs sometimes use an intermediate option and manipulate the needle by its side handle, as described by Buls et al.^(8)^ In theory, manipulation by the side handle still prevents direct hand irradiation. But it places the hand of the IR very close to the imaged plane, so that even a small accidental deviation may put the fingers in the direct beam.^(8)^

Clearly, it is important to provide IRs with all necessary information concerning the risks of CTF. But even with adequate information and training, individual attitudes towards risk vary greatly between individuals. Concerns over patient safety also play a role, since any interventional procedure presents a risk of complications. But percutaneous biopsies have an increasingly important role in modern oncology, and an initial false negative may significantly reduce the chances of patient survival.

It may be possible to enforce complete prohibitions and/or very strict rules limiting the use of CTF in some institutions. However, depending on cultural and legislative background, this may also lead to avoidance of dialogue and refusal to wear ring dosimeters. Constant and reliable monitoring of hand exposure is crucial to ensure the safe use of CTF and prevent dose escalation as a result of overconfidence.

Hand monitoring for CTF poses some technical challenges. Ring dosimeters provide only a point measurement, at a location 6–9 cm away from the finger tips. In theory, if typical dose distributions were sufficiently well known, the maximum dose could be estimated from this reading. But the dose distribution depends on hand movements, which are variable and unpredictable and occur near a high spatial gradient because the CT beam is highly collimated. So it is possible to have only the finger tips exposed in the direct beam.

For optimization of CTF practices, the ideal dosimeter would produce a complete hand dose distribution, in real‐time. This would allow IRs to optimize their own biopsy methods to reduce hand exposure, and it would be extremely useful for raising awareness of typical CTF dose rates and the associated potential for dose escalation.

At our institution, CTF is used only for the most difficult procedures, mostly lung biopsies of small lesions. When using CTF, the quick‐check method is preferred whenever possible. After initial dose assessment, the use of 20 cm long needle holders was introduced. These are currently used when real‐time manipulation becomes necessary. However, needle manipulation by the side handle, as described by Buls et al.,^(8)^ is still occasionally necessary in situations where the needle holder fails to provide sufficient grip.

We have explored the possibility of using Gafchromic XR‐QA2 self‐developing film as a complementary dosimeter to quantify hand exposure during CTF‐guided biopsies. Despite some limitations, which will be discussed in the next sections, Gafchromic film is potentially very interesting for this purpose. It changes color when exposed to ionizing radiation, allowing immediate visualization and empirical localization of the most exposed areas. The variation in optical density of the film is related to the absorbed dose, allowing comparison of two similar procedures performed in a different manner.

Gafchromic film can be digitized as well, allowing quantitative analysis of dose distribution. Absolute readings for individual monitoring purposes would require a calibration in terms of operational quantities. However, a relative dose distribution can be combined with point‐dose measurements to determine the value and the position of maximum dose. This possibility was explored in this work and the results will be presented here. The potential advantages and limitations of the method will also be discussed.

## II. MATERIALS AND METHODS

Gafchromic XR‐QA (International Specialty Products, Wayne, NJ) is a self‐developing dosimetry film developed initially as a quality assurance (QA) tool for radiology, with a dynamic range from 0.1 to 20 cGy. It has since been used, in combination with flatbed scanners, for several dosimetry applications, including measuring of CT and CBCT doses.^9^, ^10^, ^11^ The characteristics of the new Gafchromic XR‐QA2 film, relevant for kV image dose measurement, have already been extensively characterized by Giaddui et al.^(12)^

**Figure 1:**
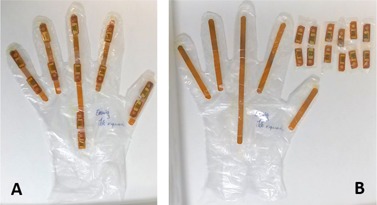
The GafTLD glove used in P7 (see Fig. 5), with both TLDs and Gafchromic strips in place (a) and after removal of the TLDs (b); the darkening of the Gafchromic strips due to irradiation is visible. Order of fingers from left to right: little finger, ring finger, middle finger, index, and thumb (gloves worn on the left hand).

For the purposes of this work, the glove developed and described by Pereira et al.^(13)^ was adapted to include five strips of Gafchromic XR‐QA2 film, approximately 7 mm wide and with the necessary length for each finger, as shown in Fig. 1. The Gafchromic strips were cut with rounded ends, so as not to tear the plastic gloves. In addition to the 11 thermoluminescent dosimeters (TLDs) initially used,^(13)^ an additional TLD was placed on the dorsal part of the hand, in the same location used by Buls et al.,^(8)^ to allow comparison of results. The completed glove (here designated GafTLD glove) contains a total of 12 dosimeters, as well as the five Gafchromic strips, as shown in Fig. 1. Only the left‐hand glove was fabricated, since previous results have shown that, for this particular IR, the left hand is consistently and significantly more exposed than the right hand during CTF‐guided biopsies.^(13)^

A total of 22 biopsies (1 bone, 2 abdomen and 19 lung or mediastinum) were performed wearing the GafTLD glove under sterile gloves. The CT scanner used for the biopsies was a four‐slice Toshiba Asteion (Toshiba, Tokyo, Japan), operated at 120 kVp, with 8 mm collimation and 0.75 s per rotation. A current intensity of 40 mA was used for all lung and mediastinum biopsies and the bone biopsy. 50 mA was used for the two abdominal biopsies. Angular beam modulation (ABM)^(14)^ is not available in this scanner.

The Gafchromic strips were cut from 25.4 cm×30.5 cm XR‐QA2 films, always along the same direction, and digitized a week after irradiation using an Epson Expression 10000XL scanner (US Epson, Long Beach, CA). The film strips were placed in the middle part of the scanner, with the yellow side facing down, and digitized in reflection mode at 72 dpi.^(12)^ A template was used so that the film could be placed always in the same position in the scanner, with marks to indicate the position of the TLD dosimeters. A nonirradiated film strip of similar dimensions was digitized as well, in a similar manner and location, to provide a control reference for calculation of net reflectance. After digitization, the red channel images were analyzed with ImageJ software (National Institute of Health, Bethesda, MA). Rectangular selections (ROIs) 4.2 mm wide were drawn along the length of the strips, and mean pixel value (MPV) profiles were obtained for these ROIs with the “plotprofile” function of ImageJ.

The TLDs used were of the Ext‐Rad type with LiF:Mg,Cu,P (TLD‐100H) detectors for the measurement of the operational quantity $H_{p}$(0.07), which is the recommended quantity for assessment of equivalent dose to local skin.^15^, ^16^ $H_{p}$(0,07) is defined as the dose equivalent to soft tissue at a depth 0.07 mm below a specified point on the body. The TLDs were calibrated in terms of $H_{p}$(0.07) using a N120 X‐ray beam incident on an ISO rod phantom.^17^, ^18^, ^19^, ^20^ The limit of detection is 0.07 mSv in terms of $H_{p}$(0.07). Transit dosimeters were used, generating mean residual dose values of 0.05 mSv. The results presented herein are net results with the background subtracted. The methodology used for calibration and read‐out of the TLDs has been described in detail elsewhere.^(13)^

During initial assessment of procedure doses and typical dose distributions,^(13)^ the use of TLD‐100H detectors calibrated in terms of $H_{p}$(0.07) allowed direct comparison with ring dosimeter measurements from routine individual monitoring. TLD‐100H detectors are often used for extremity monitoring of professionally exposed workers, and the ones we use are calibrated and read out by an approved dosimetry service and provider of individual monitoring services.

The continued use of TLD‐100H detectors in the present work aims to build on past experience, as well as facilitate comparison with previous measurements. Therefore, all TLD measurements reported will be expressed in terms of the operational quantity $H_{p}$(0.07).

TLD point measurements calibrated in the N120 beam were compared with distributions obtained from Gafchromic XR‐QA2 films, which were designed for dose measurements at low‐energy photons (20–200 kVp). The film was calibrated on the CT beam by comparison with a pencil ionization chamber reading in terms of air kerma (K). The Gafchromic XR‐QA2 film was used as a relative dosimeter to provide a proportion between the overall maximum and the reading at the location of the TLDs. A calibration is only required because Gafchromic films have a nonlinear response and therefore proportions cannot be obtained directly from pixel values of digitized images.

Small pieces of XR‐QA2 film were placed directly in the CT beam at the isocenter, together with a pencil ionization chamber (type 30009 PTW‐Freiburg, Germany), and irradiated at 120 kVp, in the same CT scanner used for the biopsies. The films were digitized a week after exposure in the manner previously described, and net reflectance (R) was calculated from mean pixel value (MPV). The fitting function used was similar to the one used by Giaddui et al.,^(12)^ of the form

(1) $$K=\frac{AR}{B-R}$$

where *A* and *B* are fitting parameters. Profiles were obtained as a function of the two parameters, A and B, and numerically integrated. Fitting was done with the Excel Solver function, obtaining the mathematical relation:

(2) $$K=\frac{79.4R}{0.482-R}$$

According to Tomic et al.,^(10)^ for XR‐QA films the difference between kerma‐based and dose‐based conversion is small and can be ignored in diagnostic applications. Giaddui et al.^(12)^ reported a calibration uncertainty of 6%–8% for Gafchromic XR‐QA2 films scanned in reflection mode at 300 dpi, decreasing to 5% at 150 dpi. This uncertainty should be even lower at 72dpi. XR‐QA2 films may be sensitive to storage temperature and humidity, as well as read‐out temperature, which are known to affect other types of Gafchromic film.^(21)^ For the EBT2 Gafchromic films used in radiotherapy, Girard et al.^(21)^ found a 1%–4% difference between calibration curves three months apart. Film autodevelopment also occurs for XR‐QA2 and was not accounted for in this work. Before digitization of the films, five blank scans were performed to warm up the lamp and scanner bed.

Measurements during clinical procedures were complemented with measurements using phantoms. A special, movable arm with attached hand was locally made from plastic materials to simulate the hand of the IR. This arm can be attached to a male RANDO phantom (The Phantom Laboratory, Salem, NY), to simulate the IR. One Alderson Lung/Chest Phantom (model RS330, Radiology Support Devices, Inc., Carson, CA) was used to simulate the patient.

**Figure 2:**
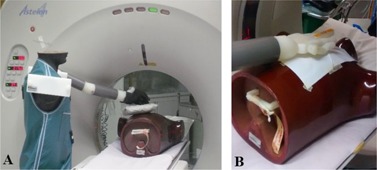
Setups used for phantom measurements: (a) phantoms and setup used to simulate a CTF‐guided biopsy, where TLDs were irradiated under controlled conditions; (b) setup used for simultaneous irradiation of TLDs and Gafchromic strips, for comparison of readings without positional uncertainty.

Two types of phantom measurements were performed. First, to determine variability of TLD readings with minor changes in positioning, a glove containing only TLDs (no Gafchromic film) was placed on the phantom hand, and the hand was positioned in such a way that the TLDs at the base of the fingers were manually aligned with the CT lasers. The irradiation was repeated five times, always taking great care to reproduce the alignment as precisely as possible. The set‐up used is shown in Fig. 2.

Second, to compare Gafchromic and TLD readings under controlled conditions, without relative movement or mechanical stress, a piece of Gafchromic film was taped to the middle finger of the plastic hand of the phantom and irradiated until the X‐ray plane was clearly identified. Strips of Gafchromic film were then cut with the same width as used in GafTLD gloves (7 mm), and marked at 1.5±0.1 cm from the edge using a marker pen, as this could be easily erased with alcohol before digitization. These strips were placed on the film taped to the phantom, with a TLD on top. Both the TLD and the mark were aligned with the irradiation plane, as shown on 2, 3 (right). Several irradiations of this setup were performed, with the irradiation parameters normally used in biopsies (120 kVp, 8 mm collimation, 40 mA and 0.75 s per rotation) and varying irradiation time (from 1.5 to 9.0 s). After digitization of the films, the mean pixel value (MPV) was obtained for the darkened part of the film and converted to dose using the mathematical relation of Eq. (2).

**Figure 3:**
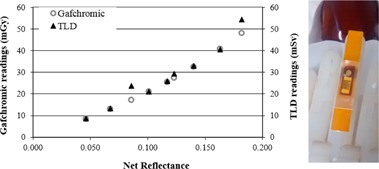
Comparison of TLD and Gafchromic readings at the same positions in phantoms. The TLDs and films were irradiated simultaneously using the setup shown in Fig. 2(b) and depicted here (on the right) under magnification. Both TLD and film readings show similar behavior, as shown on the left (graph).

## III. RESULTS & DISCUSSION

### A. Phantom measurements

**Figure 4:**
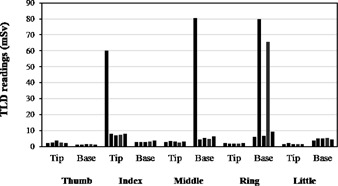
TLD readings for each finger in phantom measurements. The glove with TLDs was inserted over the articulate plastic hand and strips of Gafchromic film were used to locate the exact position of the beam before irradiation. The procedure was repeated five times, with great care to reproduce the alignment as much as possible. Clearly, when the fingers are in the beam even small differences in TLD positioning will produce very different TLD readings.

Phantom measurements performed with the setup of Fig. 2(a) show that TLD readings vary widely between irradiations for those positions which were closest to the X‐ray beam. Great care was taken in attempting to reproduce the positioning, but the results obtained for the five measurements vary extremely, as sometimes the entire TLD is directly irradiated and at other times a positional difference of 1–3 mm causes the TLD to be partially or almost completely outside the beam. These measurement results, which are presented in Fig. 4, illustrate well the effect of small positioning errors when TLDs are used very close to the irradiation plane.

The double axis plot presented in Fig. 3 shows the comparison of Gafchromic and TLD readings irradiated simultaneously, using the phantom setup of Fig. 2(b). As shown in Fig. 3, the two types of detectors used (Gafchromic and TLDs) show a similar behavior and the results seem to be numerically comparable, despite the fact that these detectors were calibrated in terms of different quantities and different conditions. The agreement between TLD and film measurements, shown in Fig. 3, suggests that it may be possible to obtain an indirect calibration of the Gafchromic XR‐QA2 film in terms of $H_{p}$(0.07). Optimizing a methodology to do this falls outside the scope of the present work.

### B. Use of GafTLD gloves during clinical procedures

**Figure 5:**
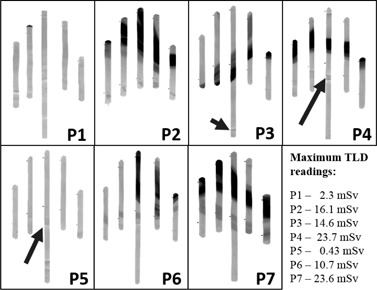
Digitized images (red channel, WL48000 WW10000) of the Gafchromic strips used during seven CTF‐guided biopsies. Window level (WL) and window width (WW) were adjusted for maximum contrast and kept constant for comparison purposes. Note that P7 corresponds to the glove shown in Fig. 1. The comparison allows an estimate of the (considerable) visual enhancement achieved through adjustment of WW and WL. The arrows (P3, P4, and P5) indicate the creases in the Gafchromic caused by bending of fingers. Order of fingers from left to right: little finger, ring finger, middle finger, index, and thumb (gloves worn on the left hand).

There were initial concerns that the Gafchromic film would not be sufficiently flexible, but preliminary testing of the gloves showed that its presence did not prevent or impair finger movement in any way. Bending of the fingers during the procedure did some damage to the Gafchromic strips, and the layers became separated around the locations of the main joints, particularly the metacarpophalangeal joints. This is clearly seen as artifacts in the digitized images (see Fig. 5), but it does not compromise dose assessment as it affects only a small portion of film and is easily recognizable as an artifact.

A total of 22 biopsies were performed wearing the GafTLD gloves under sterile gloves. All biopsies were performed by the same IR, as she is the only one using CTF at our institution. Four biopsies (one bone and three lung biopsies) were performed using the quick‐check method alone (QC procedures). In 13 biopsies (2 abdomen and 11 lung or mediastinum), the quick‐check method was combined with real‐time manipulation using a 20 cm towel clamp as needle holder (NH procedures). The remaining five biopsies (three lung and two mediastinum) required both real‐time manipulation and greater grip than is possible with the needle holder, so the IR resorted to manipulation of the needle by the side handle (SH procedures). All biopsies were performed successfully, and the IR reported no additional difficulty caused by the GafTLD gloves. The sample of biopsies is naturally biased towards real‐time manipulation, since the gloves were tested only in procedures where this was likely to happen.

TLD readings varied between 0.03 and 0.17 mSv, for QC procedures, from 0.05 to 2.3 mSv for NH procedures, and between 0.27 and 23.7 mSv, for SH procedures. XR‐QA2 film is only sensitive in the dose range from 1 to 200 mGy (0.1–20 cGy), therefore only seven procedures (P1–P7) were selected for more detailed analysis: the five SH procedures and two NH, with maximum TLD net readings of 2.3 mSv (P1) and 0.43 mSv (P5). The digitized images are shown in Fig. 5, after adjusting window level (WL) and window width (WW) for maximum contrast. The degree of visual enhancement achieved can be estimated through comparison with Fig. 1 (P7).

For the other NH and QC procedures (TLD readings between 0.03 and 1.28 mSv), the digitized images obtained are similar to the one shown for P5. It is interesting to note that, despite use of needle holders throughout the procedure, the tip of the ring finger was actually exposed in the direct beam during P1, which was an abdominal biopsy (see Fig. 5). The scatter for P1 is also higher than for other NH procedures, in good agreement with the maximum TLD reading of 2.3 mSv. This result shows that, even when needle holders are used, the fingers of the IR may be close to the X‐ray beam. The needle holder itself is 20 cm long and improvised from towel clamps, as reported by Silverman et al.^(6)^ But because the needle holder tends to slip on the needle, there may be a tendency to grip it in a different position, or hold it sideways, during some procedures.

### C. Comparison of XR‐QA2 and TLD readings during biopsies

**Figure 6:**
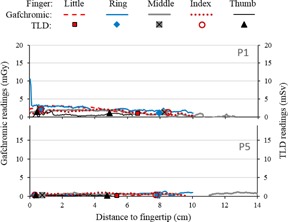
Plots of dose vs. position obtained from digitized Gafchromic strips for procedures P1 and P5 (low‐exposure NH procedures), and corresponding TLD readings for comparison. The artifacts caused by flexing were removed, therefore some dose points are missing from the middle finger profile of P5, 9–11 cm from the fingertip.

The Gafchromic readings obtained for the two low exposure NH procedures, P1 and P5, are shown in Fig. 6. Artifacts caused by mechanical damage to the film were visually identified, and the corresponding dose points deleted. Therefore, some points are missing from the middle finger dose profile of P5, around 9–11 cm from the fingertip, corresponding to the artifact shown in Fig. 5. The TLD readings are shown in the same graphs for comparison. The results obtained for the P5 procedure show that hand exposure is relatively uniform when the hand is kept distant from the X‐ray beam through the use of needle holders. For the P1 procedure, Gafchromic film readings in excess of 5 mGy were only attained at the tip of the ring finger, but exposure gradually increases towards the fingertips, suggesting increased scatter due to proximity of the direct beam.

**Figure 7:**
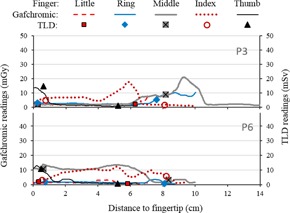
Plots of dose vs. position obtained from digitized Gafchromic strips, for procedures P3 and P6 (medium exposure SH procedures), and corresponding TLD readings for comparison. The artifacts caused by flexing were removed.

**Figure 8:**
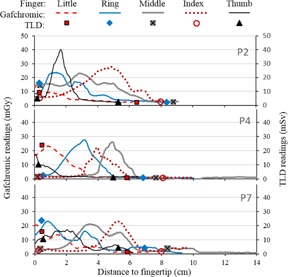
Plots of dose vs. position obtained from digitized Gafchromic strips, for procedures P2, P4, and P7 (higher exposure SH procedures), and corresponding TLD readings for comparison. The artifacts caused by flexing were removed (missing points from the middle finger dose profile of P4, 10–11 cm from the fingertip).

P3 and P6 were medium exposure procedures, with hand doses mostly below 20 mGy, as shown in the plots of Fig. 7. Hand exposure was localized during P3, and more uniform during P6. Results for the higher exposure procedures (P2, P4, and P7) are presented in Fig. 8. The variability of observed dose distributions is consistent with previous findings,^(13)^ and illustrates well the difficulty of adequate monitoring with only one point measurement (at the location of the ring dosimeter). Moreover, the fingertip is often not the most exposed area, also in agreement with previous results.^(13)^ Therefore, a single fingertip dosimeter placed over the fingernail, as sometimes used in Nuclear Medicine, would still not provide adequate monitoring for CTF‐guided procedures.

As seen in 6, 7, 8, there is generally good qualitative agreement between the dose distributions obtained from Gafchromic readings and the absolute TLD readings. A few discrepancies are obvious, both in values and position — for example, for the tips of the ring fingers in P2, P4, and P7. These may be the result of the Gafchromic strips changing position relative to TLDs because of finger movement during biopsies. TLD positions were marked on the Gafchromic while the gloves were flat on the table and, for technical reasons, the TLDs were not glued to the Gafchromic strips. As shown in Fig. 3, the effect of positioning can be significant.

### D. Comparison of results with expected readings of ring dosimeters

Table 1 shows a comparison of numerical values from TLD point measurements (back of hand, base of ring finger, and maximum overall reading) and from digitized Gafchromic strips (dose maximum and dose at base of ring finger, both absolute and expressed as a percentage of maximum). The values measured at the probable location of the ring dosimeter (base of ring finger) are very low compared with the maximum overall values. This confirms both previous results^(13)^ and the intuitive idea that, in a highly collimated field, fingertips may be far more exposed to radiation than revealed by a ring dosimeter.

It is interesting to note that doses measured at the back of the hand are similar to the median values reported by Buls et al.^(8)^ (0.18 mSv (left hand) and 0.76 mSv (right hand)), but considerably lower than their maximum values (5.98 mSv (left) and 7.34 mSv (right hand)).

**Figure 9:**
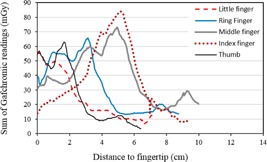
Accumulated doses. Sum of Gafchromic readings for all seven procedures, plotted as a function of distance to fingertip.

All the results presented in Table 1 are per procedure values. A ring dosimeter used for routine extremity monitoring will register exposure from several procedures: there will be a spatial average of exposure, as well. To account for this effect, we have calculated the total dose distributions by summing, for each position, the Gafchromic results for the seven procedures analyzed. These results are presented in Fig. 9. The value obtained for the ring finger, ~7 cm from the fingertip, is approximately four times lower than the overall maximum dose, which is already nearly one‐fifth of the annual dose limit, with just seven procedures.

The maximum dose value attained in Fig. 9 shows that the contribution of SH procedures towards overall annual exposure is significant, even when SH procedures are very rare. At our institution, we estimate that manipulation of the needle by the side handle is now used in less than 7% of all CTF‐guided biopsies. Considering the maximum TLD readings obtained for NH procedures (2.3 mSv, P1) and QC procedures (0.17 mSv), it seems likely that those 7% SH procedures will be responsible for most of the hand exposure in clinical practice. This is exactly why adequate hand monitoring is so important, to prevent an escalation of hand exposure through a gradual increase in frequency or duration of side handle (SH) needle manipulation.

**Table 1:** Comparison of readings from TLD measurements and digitized Gafchromic strips.

			*TLD Measurements* $H_{p}$(0.07), *mSv*			*Gafchromic Results K, mGy*		
	*Biopsy Type*	*Method*	*Max. Reading*	*Back of Hand*	*Base of Ring Finger*	*Overall Max*.	*Base of Ring Finger*	
							*Absolute*	*% Max*.
P1	Abdominal	NH	2.30	0.73	1.09	10.6	1.1	15%
P2	Lung	SH	16.13	1.04	2.09	40.1	2.5	7%
P3	Lung	SH	14.58	0.82	5.18	20.6	5.2	34%
P4	Mediastinum	SH	23.69	0.30	0.73	27.1	0.7^a^	3%
P5	Lung	NH	0.43	0.19	0.30	1.3	0.9^a^	75%^a^
P6	Lung	SH	10.69	0.32	0.82	13.4	0.8^a^	8%
P7	Mediastinum	SH	23.60	0.59	4.22	22.9	4.2	19%

^a^ Gafchromic results lower than minimum sensitivity (1 mGy) indicating increased uncertainty.

### E. Advantages and limitations of this methodology

Our GafTLD glove has some limitations, starting with the fact that it can be used only for one procedure at a time, because of mechanical damage to the Gafchromic strips. This problem might be overcome by using three small pieces of Gafchromic per finger (one piece of film for each phalanx) to avoid film bending at the joints, with each strip fixed in its place, and more resistant gloves, which would have to be custom‐made for each IR. Sterilization concerns in CTF‐guided biopsies are less than for actual surgeries; therefore, as long as the GafTLD was washable on the outside and worn under sterile gloves, its reuse should pose no greater problem than that of ring dosimeters. Its storage would require some additional precautions to avoid darkening of the XR‐QA2 film due to prolonged exposure to ambient light.

Another disadvantage of Gafchromic XR‐QA2 is its limited sensitive range, from 1 to 200 mGy. This means that, per procedure, doses can only be determined accurately for those procedures where the hands are very close to, or actually in, the X‐ray beam — which is precisely the situation we wish to avoid. On the other hand, if the same glove were to be worn during several procedures (as in routine monitoring), this minimum threshold would no longer be relevant; the XR‐QA2 would saturate at 200 mGy or 40% of the maximum annual limit. It is certainly not desirable that monthly doses should exceed 40% of the annual limit. But the potential for dose escalation with CTF is such that this is possible, when procedures are not optimized.^(22)^

The response of Gafchromic films is nonlinear and therefore requires calibration, which is another potential disadvantage. However, our results show that a basic calibration procedure is sufficient to obtain reliable qualitative dose distributions, which can be combined with a ring dosimeter, to provide one reliable point measurement for monitoring purposes.

Overall, the GafTLD gloves were well received by all elements of the interventional radiology team. A dosimeter which visibly darkens when irradiated has a great psychological impact, and this can be exploited to promote risk awareness. It leaves no doubt as to whether the hands were placed in the beam or not during the procedure. Digitized images, such as those presented in Fig. 5, can be used to explain that finger tips may be exposed to a much higher dose than the value registered by a ring dosimeter.

A nearly immediate visualization of the dose distribution is also useful for training and optimization purposes. IRs could optimize their own protection by making small adjustments in methodology and observing the effect almost immediately. From this point of view, there may be some advantages to using MOSFETs instead of TLDs, in order to have a few point measurements in real time during clinical procedures, provided their readout cycle and the X‐ray pulse are compatible.

## IV. CONCLUSIONS

The results presented here confirm the importance and potential usefulness of a hand monitoring system based on multiple point measurements or surface measurements, for interventional radiologists performing CTF‐guided procedures. Maximum exposure is not correctly assessed by ring dosimeters. Per procedure, doses at the base of the ring finger can be as low as 3%–8% of dose maximum. Considering all seven procedures analyzed in this study, the accumulated dose at the base of the ring finger was four times less than the dose maximum.

Surface hand monitoring seems feasible. Our preliminary prototype required only plastic gloves, Gafchromic XR‐QA films, and a flatbed scanner, all of which are widely available in hospitals nowadays. The IR reported no difficulty or reduction of dexterity while wearing the GafTLD gloves. Twenty‐two biopsies were successfully performed, and there was generally good agreement between dose distributions obtained with the Gafchromic strips and absolute TLD measurements, despite some positional uncertainty due to relative movement.

This prototype glove has some limitations, namely that it can be used only for one procedure, and that Gafchromic XR‐QA2 has a minimum detection threshold of 1 mGy. Nevertheless, it can be used for optimization purposes, as well as to promote risk awareness and the use of needle holders. An immediate visualization of the dose distribution has an important psychological impact, as well as providing immediate feedback.

We hope our results will prompt further research and eventually a new approach to everyday hand monitoring. CTF is a very useful imaging technique, despite the associated risks, and efficient hand monitoring would make it much safer.

## ACKNOWLEDGMENTS

The authors are grateful to all the members of the Interventional Radiology team who collaborated with this work, especially radiology technologists who assisted with CT data collection and gave valuable input regarding scanning protocols. S. Sarmento would like to thank B. Mendes for valuable help with phantom measurements and photographs. This work is funded by FEDER funds through the program Operating Competitiveness Factors ‐ COMPETE and National Funds by FCT ‐ “Fundação para a Ciência e Tecnologia” under the project PTDC/SAU‐ENB/115792/2009.
